# OsRAD17 Is Required for Meiotic Double-Strand Break Repair and Plays a Redundant Role With OsZIP4 in Synaptonemal Complex Assembly

**DOI:** 10.3389/fpls.2018.01236

**Published:** 2018-08-29

**Authors:** Qing Hu, Chao Zhang, Zhihui Xue, Lijun Ma, Wei Liu, Yi Shen, Bojun Ma, Zhukuan Cheng

**Affiliations:** ^1^State Key Laboratory of Plant Genomics and Center for Plant Gene Research, Institute of Genetics and Developmental Biology, Chinese Academy of Sciences, Beijing, China; ^2^University of Chinese Academy of Sciences, Beijing, China; ^3^College of Chemistry and Life Sciences, Zhejiang Normal University, Jinhua, China

**Keywords:** OsRAD17, meiosis, homologous recombination, synaptonemal complex, rice

## Abstract

The repair of SPO11-dependent double-strand breaks (DSBs) by homologous recombination (HR) ensures the correct segregation of homologous chromosomes. In yeast and human, RAD17 is involved in DNA damage checkpoint control and DSB repair. However, little is known about its function in plants. In this study, we characterized the RAD17 homolog in rice. In *Osrad17* pollen mother cells (PMCs), associations between non-homologous chromosomes and chromosome fragmentation were constantly observed. These aberrant chromosome associations were dependent on the formation of programmed DSBs. OsRAD17 interacts with OsRAD1 and the meiotic phenotype of *Osrad1 Osrad17* is indistinguishable from the two single mutants which have similar phenotypes, manifesting they could act in the same pathway. OsZIP4, OsMSH5 and OsMER3 are members of ZMM proteins in rice that are required for crossover formation. We found that homologous pairing and synapsis, which was roughly unaffected in *Oszip4* and *Osrad17* single mutant, was severely disturbed in the *Oszip4 Osrad17* double mutant. Similar phenotypes were observed in the *Osmsh5 Osrad17* and *Osmer3 Osrad1* double mutants, suggesting the cooperation between the checkpoint proteins and ZMM proteins in assuring accurate HR in rice.

## Introduction

Meiotic homologous recombination (HR) is initiated by the programmed formation of DNA double-strand breaks (DSBs), which was catalyzed by SPO11, a functional homolog of subunit A of an archaeal topoisomerase (TopoVIA) (Keeney et al., 1997). After resection by MRN/X complex (Mre11/Rad50/Xrs2 or Mre11/Rad50/Nbs1) and other proteins, these DSBs are processed to yield 3′ overhangs (Cao et al., 1990; Ivanov et al., 1992; Nairz and Klein, 1997). With the help of RPA (Replication Protein A), the recombinases RAD51 and DMC1 are loaded to initialize homology search and strand exchange (Bishop et al., 1992; Sung and Robberson, 1995; San Filippo et al., 2008). These early steps of HR promote homologous chromosome paring and installation of the synaptonemal complex (SC) in most organisms, including plants. The SC is a proteinaceous structure that formed between paired homologous chromosomes. Also, synapsis could occur between non-homologous chromosomes (Nairz and Klein, 1997). HR events are eventually resolved as either crossovers (COs) or non-crossovers (NCOs) in the context of the SC (Borner et al., 2004).

The ZMM proteins (ZIP1, ZIP2, ZIP3, ZIP4, MSH4, MSH5, and MER3) are meiosis-specific proteins functionally collaborating in the formation of interference-sensitive COs, which is a majority of total COs (Lynn et al., 2007). Mutants of these genes display similar phenotypes, with significantly reduced COs and high frequency of univalent formation (Ross-Macdonald and Roeder, 1994). ZMM proteins also play important roles in the assembly of the SC central element in yeast and mouse (de Vries et al., 1999; Tsubouchi et al., 2006). In contrast, the impact of ZMM proteins on synapsis appears to be minor in plants. In Arabidopsis and rice, no apparent defects in chromosome synapsis are observed in *zmm* mutants (Chelysheva et al., 2007; Higgins et al., 2008; Shen et al., 2012; Zhang et al., 2014).

RAD17 is a replication factor C (RFC)-like protein which has been demonstrated to participate in multiple processes, such as DNA damage checkpoint signaling and DSB repair (Wang et al., 2003, 2006; Budzowska et al., 2004; Heitzeberg et al., 2004). The best known function of RAD17 is recruiting the 9-1-1 complex (RAD9/HUS1/RAD1) as part of the Rad17-RFC clamp loader in response to DNA damage (Griffith et al., 2002; Zou et al., 2002; Navadgi-Patil and Burgers, 2009). In addition, RAD17 is shown to be required for loading the MRN complex at DSB site (Wang et al., 2014). The meiotic functions of RAD17 were mainly revealed in yeast. In *Saccharomyces cerevisiae*, Rad24 (the homologue of RAD17) was proved to be required for meiotic prophase arrest induced by a *DMC1* mutation, defining a meiotic recombination checkpoint (Lydall et al., 1996). In addition, Rad24 was reported to be necessary for synapsis as well as recombination template choice (Grushcow et al., 1999; Shinohara et al., 2003, 2015).

In plants, *Atrad17* mutants show increased sensitivity to the DNA-damaging agents. The *Atrad9 Atrad17* is not more sensitive to the chemicals than the single mutants, indicating that *AtRAD17* and *AtRAD9* might be epistatic (Heitzeberg et al., 2004). Moreover, AtRAD17, as the DNA damage sensor protein, is negatively regulated by a subunit of the SMC5/6 complex, SNI1 (suppressor of *npr1-1*, inducible 1), and the direct interaction between them was detected (Yan et al., 2013). Our previous study on OsRAD1 demonstrated its roles in promoting accurate meiotic DSB repair by suppressing non-homologous end joining (NHEJ) (Hu et al., 2016). NHEJ pathway involves direct ligation of the broken ends in a Ku-dependent manner and is one of the two basic strategy for DSB repair (Deriano and Roth, 2013). However, the meiotic roles of RAD17 in plants are still elusive.

Here we report the analysis of OsRAD17, the functional homolog of the mammalian RAD17 in rice. Our study indicates that OsRAD17 is required for DSB repair during meiosis in rice. In the *Osrad17* PMCs, non-homologous chromosomes associations (associations between different homologous chromosomes at pachytene and chromosome entanglements at metaphase I) existed, while homologous pairing and SC installation is roughly normal. Unexpectedly, loss of ZMM proteins in *Osrad17* mutants results in significant defects in homologous pairing and synapsis. Taken together, our data demonstrates that OsRAD17 is essential for meiotic DSB repair, and acts cooperatively with ZMM proteins in assuring SC installation in rice.

## Materials and Methods

### Plant Materials and Growth Conditions

*Osrad17-1* was isolated from sterile mutants derived by tissue culture. Other *Osrad17* mutant lines were identified from the collection of mutants induced by ^60^Co-γ-ray irradiation. The *Osspo11-1, Oscom1, Oszip4, Osmsh5, Osrad1* and *Osmer3* alleles employed in this study were previously isolated in our lab (Wang et al., 2009; Ji et al., 2012; Shen et al., 2012; Luo et al., 2013; Hu et al., 2016). Nipponbare was used as the wild type in the related experiments. All of the plants were grown in paddy fields in Beijing (China) or Sanya (Hainan Province, China) during the natural growing season.

### Map-Based Cloning of *OsRAD17*

In a screen for rice meiotic mutants, we identified three mutant lines that segregated 3:1 for fertile and sterile plants, indicating that they are belonging to the single recessive mutation.

A map-based cloning approach was adopted to isolate the target gene. We crossed *Osrad17-1, Osrad17-2*, and *Osrad17-3* heterozygous mutant plants with the *indica* rice variety Zhongxian3037 to produce the mapping populations. Using sterile plants that segregated in F2 population (28 plants for *Osrad17-1*, 22 for *Osrad17-2, and* 20 for *Osrad17-3*), a linked marker to the sterile phenotype was found for all the three populations. Then we carried out fine mapping with additional sterile F2 and F3 plants to pinpoint the target gene within a 200-kilobase region. According to the MSU Rice Genome Annotation Project Database and Resource^1^, we found a candidate gene (*LOC_Os03g13850*) annotated as the putative cell cycle checkpoint protein RAD17. Sequencing of this gene in the three mutant lines showed that they all had mutation sites in the coding region.

Indel (insertion–deletion) markers used for mapping were designed based on the sequence differences between *indica* variety 9311 and *japonica* variety Nipponbare according to the data published^2^. Primer sequences used were listed in **Supplementary Table S1**.

### Full-Length cDNA Cloning of *OsRAD17*

Total RNA extraction from rice young panicles was conducted using the TRIzol reagent (Invitrogen). Reverse transcription was performed with primer Adaptor-T (18) using the superscript III RNaseH reverse transcriptase (Invitrogen). For RACE, 3′-Full RACE Core Set with PrimeScript^TM^ RTase (TaKaRa) and 5′-Full RACE Kit with TAP (TaKaRa) were used to identify the 3′ end and 5′ end of the cDNA, respectively. PCR using primers RO-F and RO-R was performed to amplify the open reading frame. Then the products were cloned into the PMD18-T vector (TaKaRa) and sequenced. The sequences were then spliced together to obtain the full-length cDNA sequence.

### Quantitative RT-PCR Assay

Total RNA was extracted individually from roots, leaves, internodes and young panicles (5–7 cm long) of Nipponbare, and was reverse-transcribed into cDNA. Real-time RT-PCR analysis was performed using the Bio-Rad CFX96 real-time PCR instrument and EvaGreen (Biotium). All PCR experiments were conducted using 40 cycles of 95°C for 10 s, 60°C for 30 s and were performed in triplicate. Gene-specific primers (17RT-F/17RT-R) and standard control primers (Actin-F/Actin-R) were listed in **Supplementary Table S1**.

### Cytology

Preparations of rice PMCs were performed as described (Shen et al., 2012). The primary antibodies used in immunofluorescence were anti-OsREC8, anti-ZEP1, anti-OsCOM1, anti-OsDMC1 and anti-γH2AX (Wang et al., 2010, 2016; Shao et al., 2011; Ji et al., 2012). FISH analysis was conducted according to Yang et al. (2016). Original images were captured under Zeiss A2 fluorescence microscope with a micro CCD camera.

### Yeast Two-Hybrid (Y2H) Assay

The Y2H assays were performed using the Matchmaker Gold Yeast Two-Hybrid system (Clontech). The full length CDS of OsRAD17 and 9-1-1 proteins were cloned into pGADT7 and pGBKT7 to generate AD and BD recombinants. Quadruple dropout (QDO) selection medium with aureobasidin A and the chromogenic substrate X-a-Gal was used to verify the interaction and double dropout (DDO) selecting medium (SD -Leu -Trp) to confirm the successful transformation.

## Results

### Characterization of *Osrad17* Mutant Alleles

**FIGURE 1:**
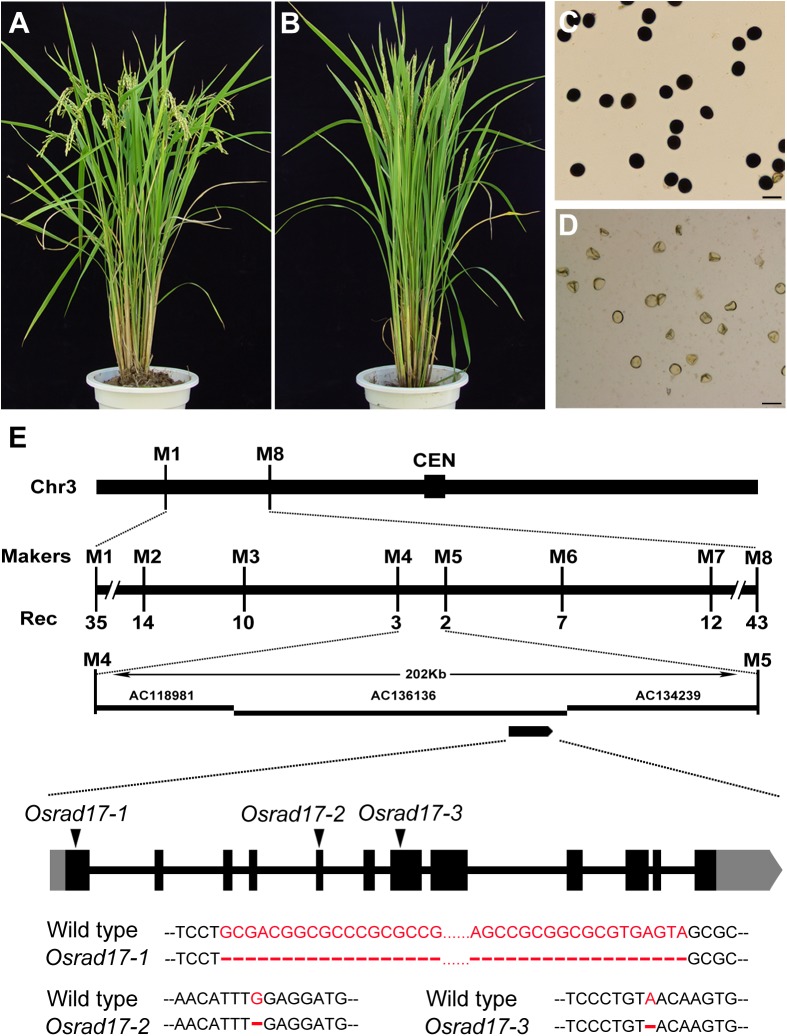
Characterization of the *Osrad17* mutant and map-based cloning of *OsRAD17* gene. **(A)** Wild type plant. **(B)** *Osrad17* plant. **(C)** Pollen grains of wild type. Bars, 50 μm. **(D)** Pollen grains of *Osrad17* mutant. Bars, 50 μm. **(E)** Map-based cloning of *OsRAD17* gene and gene structure. CEN, centromere. Rec, the number of recombinants. Coding regions are shown as black boxes, and untranslated regions are shown as gray boxes. The triangles indicate the mutated sites and detailed mutations were listed below.

In a screen for rice meiotic mutants, we obtained three mutant lines allelic for disruption in *LOC_Os03g13850* through map-based cloning (**Figure 1**). This gene was named *Oryza sativa RAD17* (*OsRAD1*7), based on the homology of the protein sequence (see below) and the three mutants were *Osrad17-1* (Nipponbare), *Osrad17-2* (Yandao 8), and *Osrad17-3* (Yandao 8), respectively. *Osrad17-1* mutant exhibited normal vegetative growth but complete sterility (**Figures 1A–D**). I_2_-KI staining showed that the pollen grains were completely non-viable in the mutant. Pollinating the mutant flowers with wild type pollen did not set any seeds, suggesting that the mutant is both male and female sterile.

Sequencing of the *OsRAD17* gene in the *Osrad17-1* mutant revealed a 64 bp deletion in the first exon (**Figure 1E**), which resulted in frame shift and premature stop codon formation. In *Osrad17-2* and *Osrad17-3*, a single nucleotide deletion occurred in the fifth and seventh exon, respectively, leading to frame shift and premature stop codon. We selected *Osrad17-1* for most subsequent studies.

We obtained a 2740 bp full-length cDNA of *OsRAD17* by performing rapid amplification of cDNA ends (RACE), which encoded a protein of 620 amino acids. The result of PSI-BLAST search in public databases revealed that OsRAD17 shared significant similarity with RAD17 protein of Arabidopsis (43% identity and 60% similarity), human (29% identity and 46% similarity) and fission yeast (27% identity and 38% similarity). A Reciprocal Best BLAST further confirmed that the protein that we isolated was the closest relative of RAD17 in rice. Multiple sequence alignment of OsRAD17 with its orthologs showed that the RAD17 proteins were highly conserved, especially within the AAA-ATPase domain (**Supplementary Figure S1**).

Quantitative RT-PCR showed that *OsRAD17* was expressed as early as the seedling stage. In adult-stage rice, *OsRAD17* was detectable not only in young panicles but also in leaves, roots, and internodes (**Supplementary Figure S2**).

### Non-homologous Chromosome Associations and Fragmentations Shown in *Osrad17*

**FIGURE 2:**
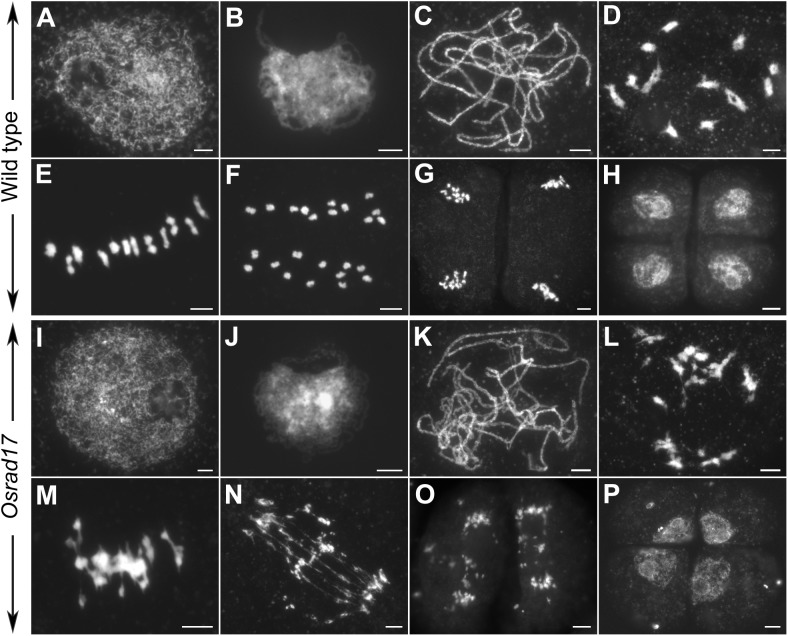
Chromosome spreads of PMCs in wild type and *Osrad17*. Chromosomes were stained with DAPI. **(A,I)** Leptotene. **(B,J)** Zygotene. **(C,K)** Pachytene. **(D,L)** Diakinesis. **(E,M)** Metaphase I. **(F,N)** Anaphase I. **(G,O)** Anaphase II. **(H,P)** Tetrad. Bars, 5 μm.

**FIGURE 3:**
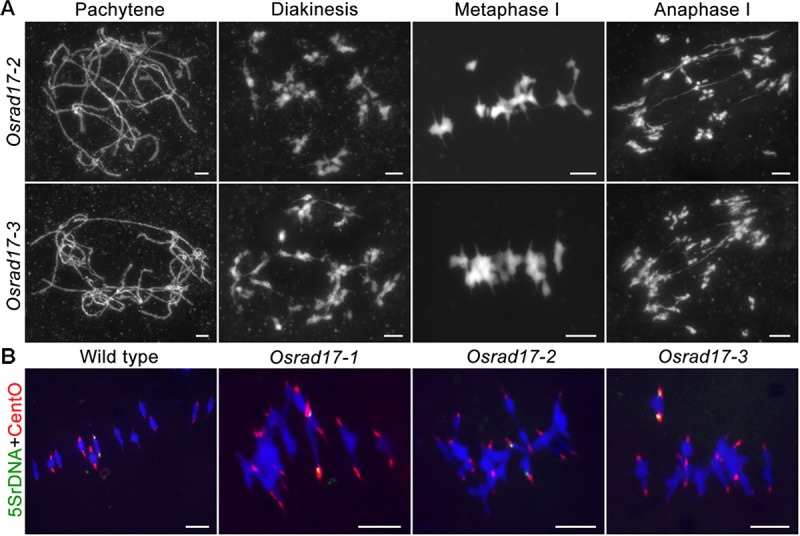
Non-homologous associations in different *Osrad17* alleles. **(A)** Chromosome spreads of PMCs in *Osrad17-2* and *Osrad17-3* at different meiotic stages. **(B)** FISH analysis of the metaphase I chromosome interactions in wild type and three *Osrad17* mutants. Bars, 5 μm.

To determine whether pollen abortion resulted from the defects in male meiosis, we investigated chromosome behavior in PMCs by 4′,6-diamidino-2-phenylindole (DAPI) staining. By comparing *Osrad17-1* with the wild type, we found that *Osrad17-1* chromosomes behaved in a similar way to those of wild type from leptotene to zygotene (**Figures 2A,B,I,J**). However, aberrations were observed thereafter. At pachytene, *Osrad17-1* chromosomes presented as thick threads and synapsed chromosomes were visible, just like the wild type (**Figures 2C,K**). But associations between non-homologous chromosomes were found among *Osrad17-1* synapsed chromosomes (**Figure 2K**). At diakinesis and metaphase I, wild type PMCs had twelve condensed bivalents (**Figures 2D,E**), while *Osrad17-1* exhibited chromosome aggregations (**Figures 2L,M**). Homologous chromosomes separated precisely at anaphase I and chromatids separated at the second meiotic division, producing tetrads in wild type (**Figures 2F–H**). In contrast, extensive chromosome bridges and fragments were observed in *Osrad17-1*, which generated abnormal tetrads with micronuclei (**Figures 2N–P**). The chromosome behaviors of *Osrad17-2* and *Osrad17-3* showed the same meiotic defects with *Osrad17-1* (**Figure 3A**).

To explore the nature of the chromosome aggregations during metaphase I in the mutants, we performed fluorescent *in situ* hybridization (FISH) experiments using 5S rDNA and CentO probes (**Figure 3B**). The 5S rDNA was located on the centromere-proximal region of chromosome 11 and CentO is a molecular marker for all rice centromeres. In wild type PMCs, 12 bivalents were aligned in the middle of the cell. There are two CentO signals on each bivalent and two 5S rDNA signals on chromosome 11. However, on the chromosome aggregations of the mutants, more than two CentO signals were observed, indicating that these aggregations contain more than one pair of homologous chromosomes. Thus, the chromosome aggregations were the associations among non-homologous chromosomes.

### Meiotic Defects in *Osrad17* Are Dependent on Programmed DSB Formation

**FIGURE 4:**
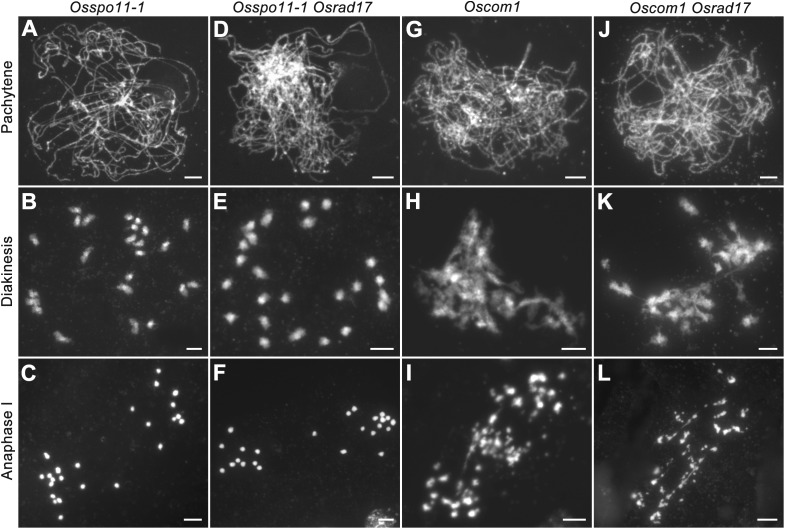
Genetic analysis of *OsRAD17* with *OsSPO11-1* and *OsCOM1*. **(A–C)** Chromosomes behaviors in *Osspo11-1* at corresponding meiotic stages. **(D–F)** Chromosomes behaviors in *Osspo11-1 Osrad17* was similar to *Osspo11-1*. **(G–I)** Chromosomes behaviors in *Oscom1* at corresponding meiotic stages. **(J–L)** Chromosomes behaviors in *Oscom1 Osrad17* was similar to *Oscom1*. Bars, 5 μm.

To determine whether the chromosomal abnormalities in *Osrad17* were acquired during meiotic DSB repair, we constructed the *Osspo11-1 Osrad17* double mutant. The *Osspo11-1* mutants display intact univalents that are randomly distributed due to the absence of meiotic DSBs (**Figures 4A–C**). Cytogenetic analysis of *Osspo11-1 Osrad17* PMCs revealed univalents in metaphase I with no evidence of chromosome aggregations or fragmentations (**Figures 4D–F**). Thus, the meiotic defects in *Osrad17* are related to the repair of the OsSPO11-dependent programmed DSBs. OsCOM1 is required for processing of meiotic DSBs. Lack of OsCOM1 leads to abolished HR (Ji et al., 2012). In the *Oscom1* mutant, homologous chromosomes failed in synapsis, and non-homologous associations were observed (**Figures 4G–I**). The meiotic phenotype of *Oscom1 Osrad17* double mutant mimicked that of the *Oscom1* single mutant, as synapsis in *Oscom1 Osrad17* was inhibited due to the mutation of *OsCOM1* (**Figures 4J–L**) (**Supplementary Figure S3**). This suggested that OsRAD17 may functions downstream of OsCOM1 in rice meiosis.

### OsRAD17 Functions Together With OsRAD1 on Meiotic DSB Repair

**FIGURE 5:**
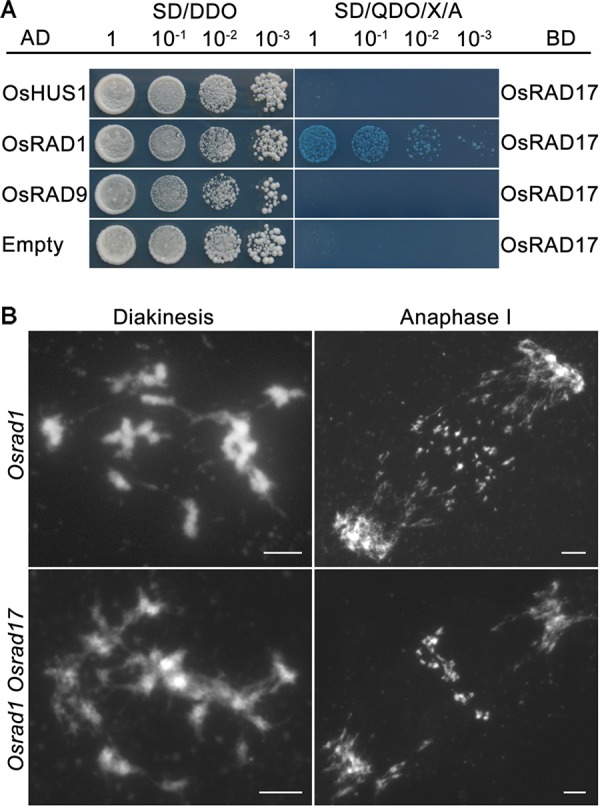
Physical and genetic interaction between OsRAD17 and OsRAD1. **(A)** OsRAD17 interacts with OsRAD1 in yeast two-hybrid assays. **(B)** The *Osrad1 Osrad17* double mutant shows similar chromosome behaviors to those of the single mutant at corresponding meiotic stages. Bars, 5 μm.

The intimate relationship between RAD17 and the 9-1-1 complex has been well demonstrated by numerous researches. Our previous studies proved that the 9-1-1 complex was involved in meiotic DSB repair (Che et al., 2014; Hu et al., 2016). To determine if OsRAD17 associated with the 9-1-1 for DSB repair during rice meiosis, we conducted yeast two-hybrid assays and genetic analysis. Yeast two-hybrid assays revealed a direct interaction between OsRAD17 and OsRAD1 (**Figure 5A**). No interaction between OsRAD17 and OsRAD9 or OsHUS1 was detected. This suggests that OsRAD17 may load the 9-1-1 complex by interacting with OsRAD1 in rice meiosis.

To further verify the genetic relationship between *OsRAD17* and *OsRAD1*, we constructed the *Osrad1 Osrad17* double mutant. The double mutant exhibited similar meiotic phenotype with the single mutant, also indicating that they could act in the same meiotic DSB repair pathway (**Figure 5B**).

### Homologous Chromosome Pairing and SC Assembly Are Disturbed in the *Oszip4 Osrad17* Double Mutant

**FIGURE 6:**
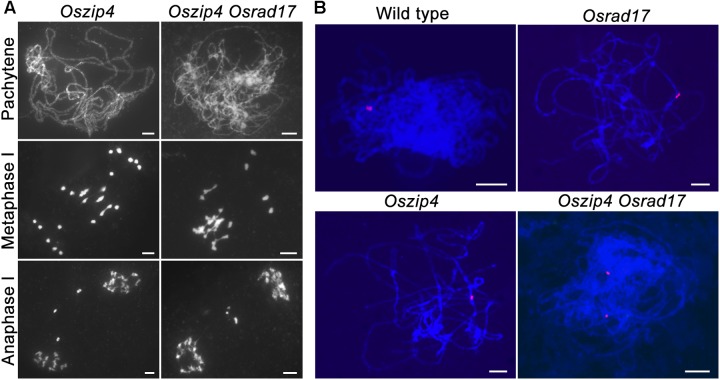
OsRAD17 and OsZIP4 are redundantly required for homologous pairing. **(A)** Chromosomes behaviors in *Oszip4* and *Oszip4 Osrad17* at corresponding meiotic stages. **(B)** Localization of 5S rDNA signals in wild type, *Osrad17, Oszip4* and *Oszip4 Osrad17* PMCs during pachytene. Bars, 5 μm.

To explore the relationship between ectopic chromosome interactions in *Osrad17* and interference-sensitive COs, we generated the *Oszip4 Osrad17* double mutant. Loss of OsZIP4 results in the reduction of bivalents and appearance of univalents due to reduced CO formation (Shen et al., 2012). *Oszip4 Osrad17* showed a mixture of both univalents and chromosome aggregations at metaphase I (**Figure 6A**), indicating that aberrant chromosome associations in *Osrad17* arise independently from the ZMM proteins-mediated pathway. We also observed a similar phenotype in the *Osmsh5 Osrad17* double mutant (**Supplementary Figure S4**).

Moreover, we found that homologous pairing seemed to be severely disturbed in the *Oszip4 Osrad17* mutant at pachytene stage (**Figure 6A**). This is quite interesting, considering that both of the single mutant displayed roughly normal pairing. We further performed chromosome-specific FISH analysis using 5S rDNA (**Figure 6B**). At early pachytene, two adjacent red signals, representing the closely paired homologous chromosomes, were observed in wild type, *Osrad17* and *Oszip4*. However, 85.7% of the *Oszip4 Osrad17* cells (*n* = 35) had two separated 5S rDNA signals, showing that the chromosome 11 were partially separated. This observation indicates that OsRAD17 and OsZIP4 act cooperatively to promote homologous pairing.

**FIGURE 7:**
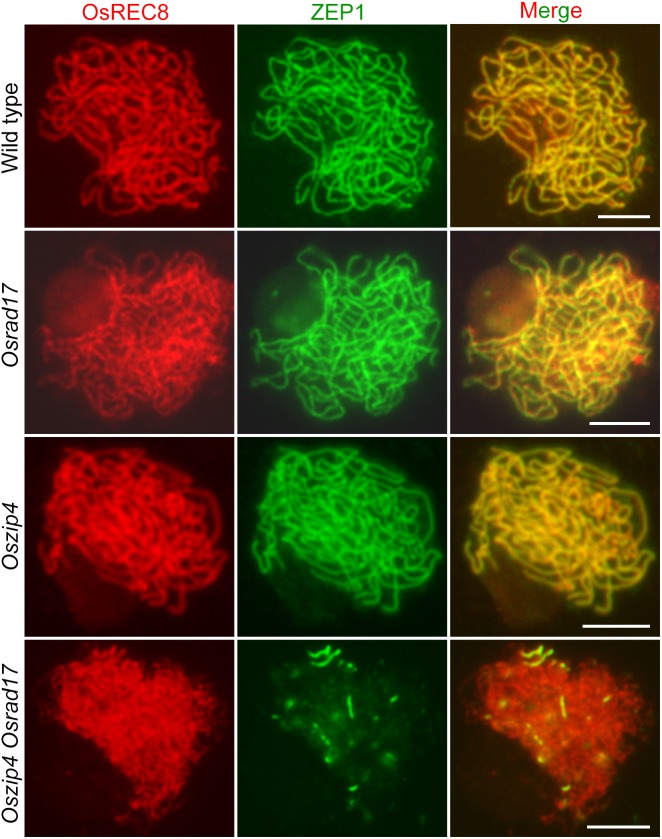
OsRAD17 plays a redundant role with OsZIP4 in synaptonemal complex assembly. OsREC8 signals (red) were used to indicate the chromosomes. Immunostaining for ZEP1 (green) in wild type, *Osrad17, Oszip4* and *Oszip4 Osrad17* at pachytene are shown. Bars, 5 μm.

During meiosis, homologous pairing and synapsis are closely related. Considering abnormal homologous pairing in *Oszip4 Osrad17*, we next wanted to determine the synaptonemal complex assembly in this double mutant. We examined the localization of ZEP1 and OsREC8 in *Osrad17* as well as *Oszip4*. ZEP1 is a central element of SC and a perfect maker to indicate the extent of synapsis in rice (Wang et al., 2010). OsREC8 is the meiosis-specific cohesin, signal of which predicts the axial element of SC (Shao et al., 2011). Localization of OsREC8 and ZEP1 in *Oszip4* was indistinguishable from the wild type and ZEP1 signal in *Osrad17-1* could be detected along almost the entire chromosomes, with the exception of a few discontinuities (**Figure 7**). This suggests that SC assembly is roughly normal in these mutants. However, localization of ZEP1 was rare in *Oszip4 Osrad17* (**Figure 7** and **Supplementary Figure S5**). The percentage of synapsis in most *Oszip4 Osrad17* PMCs was less than 25% (*n* = 30). This proved that OsRAD17 plays a redundant role with OsZIP4 in SC assembly. We further detected immunolocalization of ZEP1 in *Osmsh5 Osrad17*, finding the similar incomplete SC formation (**Supplementary Figures S5, S6**). These data suggest an important role for OsRAD17 in promoting SC installation in the absence of ZMM proteins. To investigate if the 9-1-1 complex is also involved in regulating SC installation, we examined the localization of ZEP1 in *Osmer3 Osrad1* by immunofluorescence and found incomplete ZEP1 signal at pachytene (**Supplementary Figures S5, S6**), indicating that OsRAD1 and OsMER3 are also redundantly necessary for SC assembly. These results together manifested that there might be a mechanism for homologous pairing and synaptonemal complex assembly requiring the cooperation between the checkpoint proteins and ZMM proteins.

### HR Events in *Oszip4 Osrad17* Double Mutant

**FIGURE 8:**
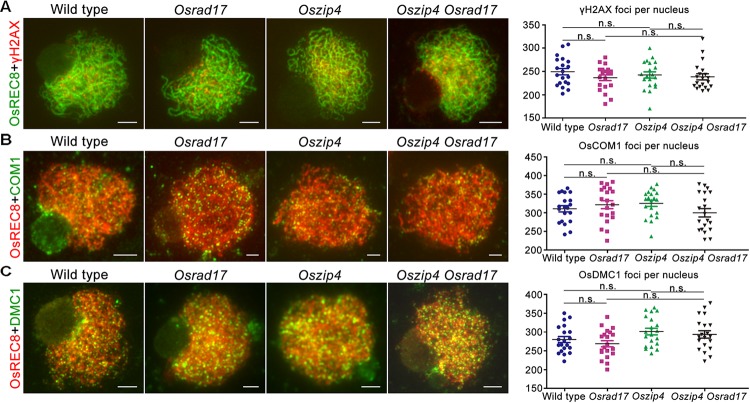
Analysis of HR events in *Oszip4 Osrad17* double mutant. **(A)** Immunofluorescent investigation of γH2AX signals on the meiotic spreads of wild type, *Osrad17, Oszip4* and *Oszip4 Osrad17*. Bars, 5 μm. **(B)** Immunofluorescent investigation of OsCOM1 signals on the meiotic spreads of wild type, *Osrad17, Oszip4* and *Oszip4 Osrad17*. Bars, 5 μm. **(C)** Immunofluorescent investigation of OsDMC1 signals on the meiotic spreads of wild type, *Osrad17, Oszip4* and *Oszip4 Osrad17*. Bars, 5 μm. Scatter plot of foci per nucleus were presented (*n* = 20). n.s., no significant differences, *P* > 0.05 in two-tailed Student’s *t*-tests.

To verify the cause of defects in chromosome paring and synapsis, we monitored HR in *Oszip4 Osrad17* meiosis (**Figure 8**). γH2AX is the phosphorylated form of H2AX, the DSB repair specific histone variant accumulating at the sites of DSBs (Rogakou et al., 1999). Therefore, DSB formation is able to be inferred from the γH2AX foci. Our analysis revealed similar localization of γH2AX in wild type, *Osrad17, Oszip4* and *Oszip4 Osrad17* (**Figure 8A**). This suggests that DSB formation is unaffected in *Oszip4 Osrad17*.

We next investigated the localization of OsCOM1 and OsDMC1 in *Oszip4 Osrad17* mutants (**Figures 8B,C**). OsCOM1 and OsDMC1 have been proved to be essential for DSB end resection and strand-exchange, respectively (Ji et al., 2012; Wang et al., 2016). Based on our observation, there was no significantly difference in the localization of OsCOM1 as well as OsDMC1 among wild type, *Osrad17, Oszip4* and *Oszip4 Osrad17*. Thus, despite the defective homologous pairing and synapsis, HR is normally initiated in the *Oszip4 Osrad17* double mutant.

## Discussion

### OsRAD17 Is Required for Meiotic DSB Repair in Rice

Studies in yeast and mammals have elucidated the multiple functions of RAD17, a well-known checkpoint component (Lydall et al., 1996; Grushcow et al., 1999; Zou et al., 2002). Here, we isolated the RAD17 homolog in rice. Loss of OsRAD17 results in abnormal chromosome associations and fragmentations in PMCs. The *Osrad17* meiotic phenotype depends on programmed DSB formation, indicating the role of OsRAD17 in DSB repair. This phenotype resembles that of the 9-1-1 mutants in rice. In *Oshus1* and *Osrad1* mutant, DSB-dependent chromosome associations and fragments were also observed (Che et al., 2014; Hu et al., 2016). OsRAD17 can interact with OsRAD1 as revealed by yeast two-hybrid assays. In addition, the phenotype of *Osrad1 Osrad17* double mutant cannot be distinguished from that of each single mutant, indicating that they might function in the same pathway. Thus, we propose that OsRAD17 may participate in meiotic DSB repair by loading the 9-1-1 complex in rice.

### OsRAD17 May Be Involved in DSB Repair Pathway Choice

Mitotic cells employ two basic strategies for DSB repair: HR and classical non-homologous end-joining (C-NHEJ), which involves direct ligation of the broken ends (Deriano and Roth, 2013). In *Arabidopsis*, the *Atrad17* mutant shows no significant increase in DNA damage, indicating NHEJ pathway might be sufficient to repair DNA damage (Yan et al., 2013). During meiosis, C-NHEJ competes with HR and creates de novo mutations in the gametes. Thus, this pathway should be restricted during meiotic DSB repair. Our previous study showed that C-NHEJ mediated by Ku70 accounted for most of the ectopic associations in *Osrad1*. In the *Osku70 Osrad1* PMCs, chromosome aggregations were partially suppressed compared with *Osrad1* (Hu et al., 2016). Thus, we speculated that OsRAD17 might participate in the DSB repair pathway choice during meiosis. Recent studies in yeast demonstrated the function of 9-1-1 during DNA resection, which affects DNA repair pathway choice (Blaikley et al., 2014; Ngo et al., 2014). Further studies are needed to test whether OsRAD17, together with 9-1-1 complex, functions through extensive DNA end resection in rice meiosis.

In budding yeast, the DNA damage response clamp 9-1-1 promotes ZMM proteins to take part in crossover formation and synaptonemal complex assembly (Shinohara et al., 2003, 2015). In the *rad24* mutant, Zip1 elongation is defective. In the *zip4* mutant, the synaptonemal complex protein Zip1 fails to polymerize along chromosomes (Tsubouchi et al., 2006). In mice carrying a disruption in MutS homolog Msh5, aberrant chromosome synapsis was observed (de Vries et al., 1999). These studies proved the involvement of both checkpoint proteins and ZMM proteins in SC formation. However, in plants, synapsis is almost normal in mutants related to these genes. Unexpectedly, the combination of mutations in both checkpoint proteins and ZMM proteins severely disrupted homologous pairing and SC installation. We propose that the repair of most DSBs by HR in *Osrad17* is adequate for the homologous pairing and synapsis. However, more detailed studies are required to verify this speculation.

### Functional Divergence of RAD17 in Different Plants

In Arabidopsis, AtRAD17 involves in DNA damage repair, which is negatively regulated by SMC5/6 complex (Heitzeberg et al., 2004; Yan et al., 2013). However, the mutation in *Atrad17* shows very weak effects on meiosis. The similar situations were observed for genes involved in anti-COs pathways. For example, FIGL1 and its partner FLIP in *Arabidopsis* function in limiting the number of COs. Mutants of these genes did not cause obvious defects in meiosis (Girard et al., 2015; Fernandes et al., 2018). However, disruptions of *MEICA1*, the homolog of *FLIP*, and *OsFIGNL1* led to non-homologous chromosome associations and fragmentations (Hu et al., 2017; Zhang et al., 2017). The difference in genome organization may cause the functional divergence of these proteins in meiosis (Lambing and Heckmann, 2018). Compared with *Arabidopsis*, rice has a bigger genome and contains more repetitive sequences.

## Author Contributions

ZC and QH conceived the original screening and research plans. YS and BM supervised the experiments. QH, CZ, and ZX performed most of the experiments. QH, LM, and WL designed the experiments and analyzed the data. QH conceived the project and wrote the article with contributions of all the authors. ZC supervised and complemented the writing.

## Conflict of Interest Statement

The authors declare that the research was conducted in the absence of any commercial or financial relationships that could be construed as a potential conflict of interest.

## Footnotes

1 http://rice.plantbiology.msu.edu/

2 http://www.ncbi.nlm.nih.gov
