# Supplementary material for: OsRAD17 Is Required for Meiotic Double-Strand Break Repair and Plays a Redundant Role With OsZIP4 in Synaptonemal Complex Assembly
Source: Front Plant Sci. 2018 Aug 29;9:1236. doi: 10.3389/fpls.2018.01236 (PMC6123563; doi:10.3389/fpls.2018.01236)
Supplement: Supplementary file 1 [file Data_Sheet_1.PDF]

Supplemental files

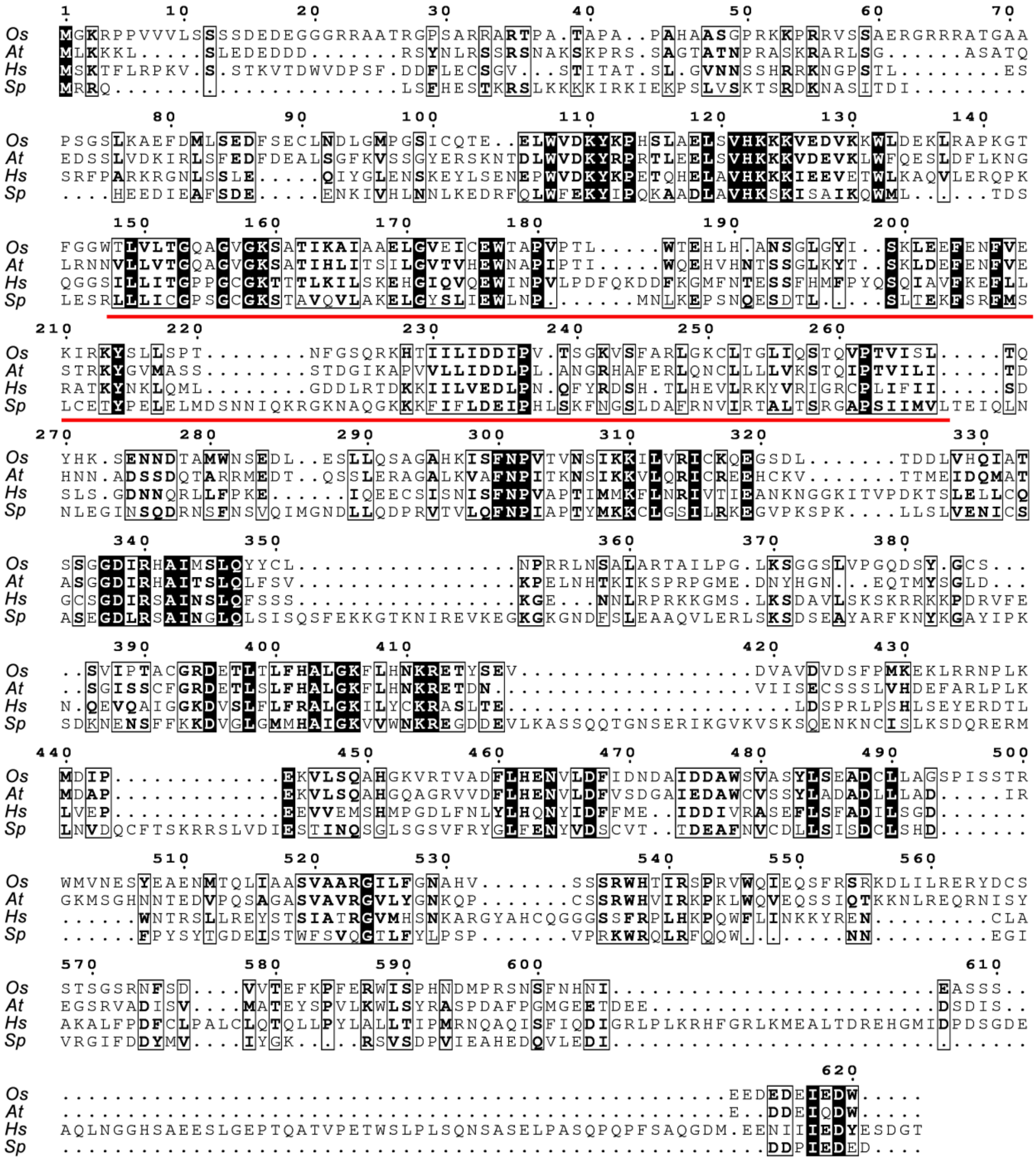

FIGURE S1 Multiple sequence alignment of OsRAD17 homologs. *Os*, *Oryza sativa*; *At*, *Arabidopsis thaliana*; *Hs*, *Homo sapiens*; *Sp*, *Schizosaccharomyces pombe*. Identical amino acids are shaded in black and the conserved AAA-ATPase domain is indicated with the red lines.

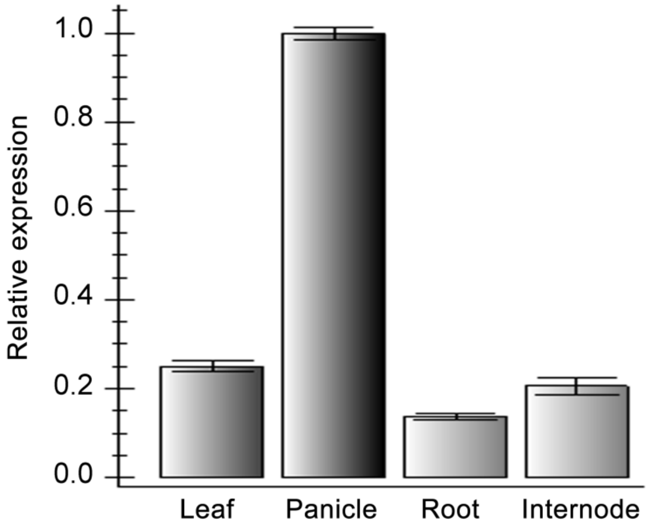

FIGURE S2 The expression analysis of *OsRAD17***.** Expression analysis of *OsRAD17* in different tissues by quantitative RT-PCR. *Actin* was used as the endogenous control. The error bars represent the SE of mean values in three biological replicates.

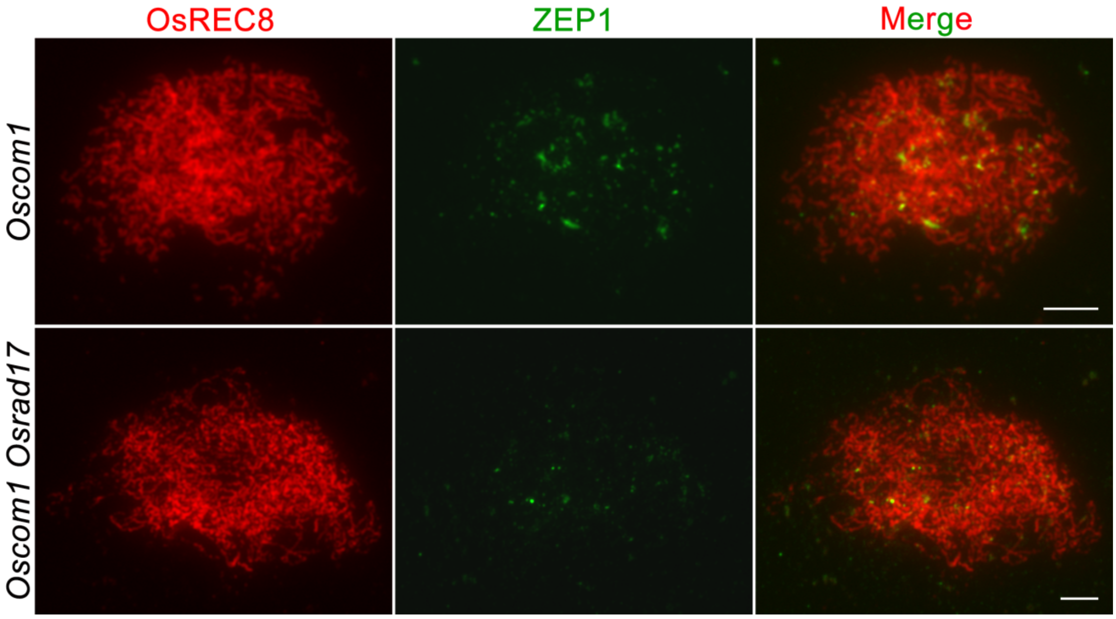

FIGURE S3 Synapsis is defective in *Oscom1* and *Oscom1 Osrad17*. OsREC8 signals (red) were used to indicate the chromosomes. Bars, 5 µm.

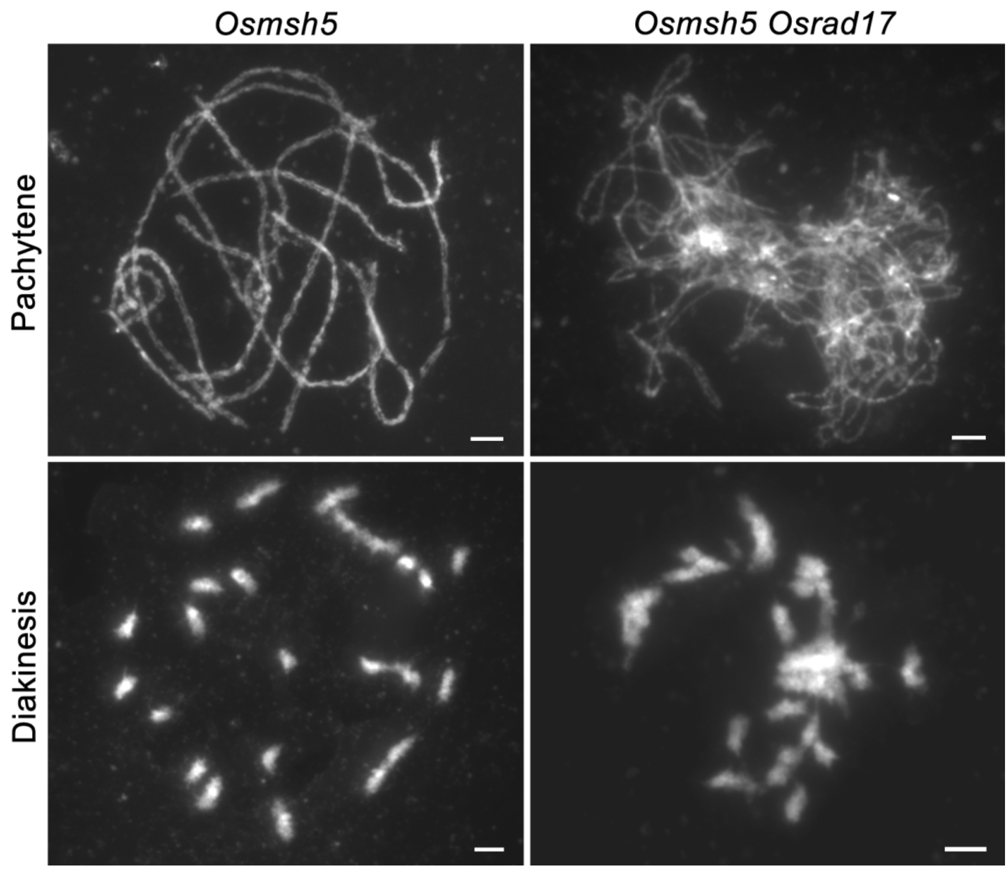

FIGURE S4 Chromosomes behaviors in *Osmsh5* and *Osmsh5 Osrad17* at corresponding meiotic stages. Nonhomologous chromosomes associations occur and synapsis is disturbed in the *Osmsh5 Osrad17* double mutant. Bars, 5 μm.

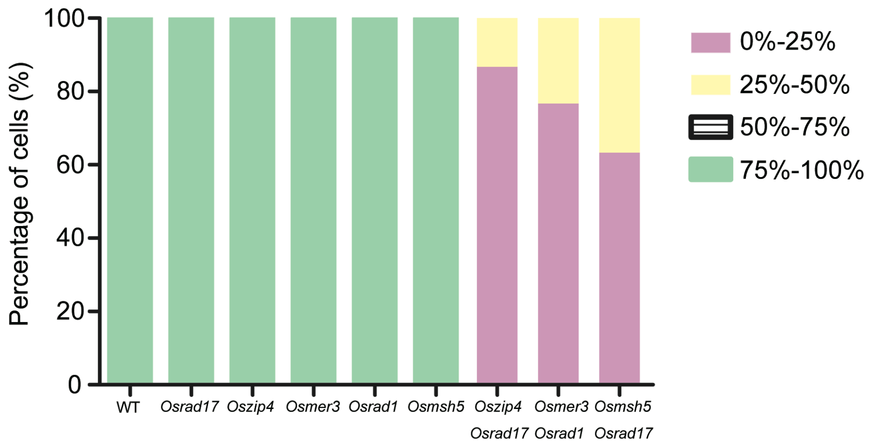

FIGURE S5 Distributions of PMCs with different synapsis percentage in wild type and mutants (n=30).

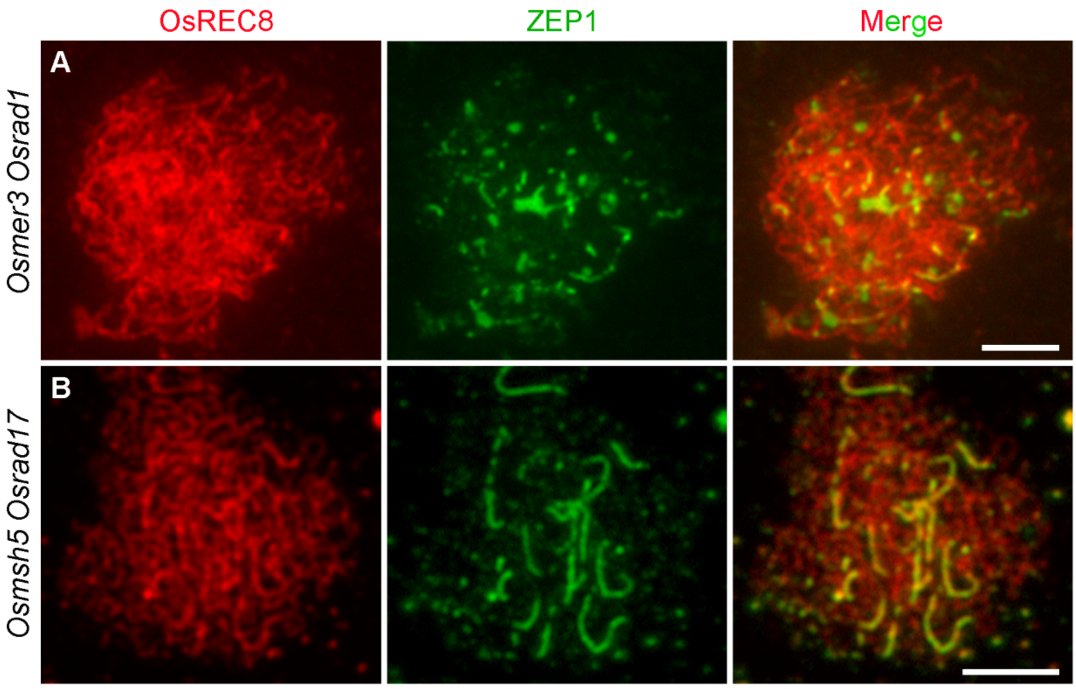

FIGURE S6 Localization of ZEP1 in *Osmer3 Osrad1* and *Osmsh5 Osrad17*. (A) Localization of ZEP1 in *Osmer3 Osrad1*. (B) Localization of ZEP1 in *Osmsh5 Osrad17*. Bars, 5 μm.

TABLE S1 Primers used in this study

Primer name	Sequence
Mapping-1F	CGGACAACGGCATGTCAAATC
Mapping-1R	TTTCTGCGGCGTTTGTTGG
Mapping-2F	CCTGTTCACACCAACAATCC
Mapping-2R	GTAACACGTACGTATTGATG
Mapping-3F	GGCATTCGCGAAGAGGGGAAG
Mapping-3R	GCGCCCCGTCCCCCATGCGGT
Mapping-4F	GTGCTAATGAGTCGCTTGG
Mapping-4R	TCAAAGTCAATGTTGTCATA
Mapping-5F	TCATGCTGGCGTTAACTTTGG
Mapping-5R	CATCTTATAACAACAAAATTG
Mapping-6F	CCGGAACAGATCATCAAG
Mapping-6R	CGAACCAGCACTCATAGC
Mapping-7F	GCCCCTTGTTCCCTGCATAT
Mapping-7R	GAACCTCGTCTCGCTCGTGG
Mapping-8F	ACGCTAGAGACTAATGGCTT
Mapping-8R	CTGCTCAGGCATTAAAAAGG
17RT-F	TGCAATGTGGAACTCTGAGG
17RT-R	GCATGTCTGATATCACCTCCG
Actin-F	CTGACAGGATGAGCAAGGAG
Actin-R	GGCAATCCACATCTGCTGGA
Adaptor-T(18)	CTGATCTAGAGGTACCGGATCCTTTTTTTTTTTTTTTTTT
RAD17-3RACE-1	GTACATCTGGTTCTCGCAATT
RAD17-3RACE-2	ATGATATGCCTAGAAGTAAC
RAD17-5RACE-1	TGACATATAGATCCTGGCATGC
RAD17-5RACE-2	GCTACTCACGCGCCGCGGCTTC
RO-F	ATGGGGAAGCGGCCGCCGGT
RO-R	TCACCAATCTTCTATCTCAT
OsRAD17-PGADT7-F	CATATGATGGGGAAGCGGCCGCCGGT
OsRAD17-PGADT7-R	GTCGACTCACCAATCTTCTATCTCAT
OsRAD17-PGBKT7-F	CATATGATGGGGAAGCGGCCGCCGGT
OsRAD17-PGBKT7-R	GTCGACTCACCAATCTTCTATCTCAT
